# A case of suspected peritoneal thermal injury complicated by septic shock following infusion of perceptibly hot peritoneal dialysate

**DOI:** 10.1186/s12882-026-05314-0

**Published:** 2026-08-31

**Authors:** Weizhong Liang, Nan Feng, Xiaozhong Li, Xinting Lu

**Affiliations:** 1 Department of Nephrology, The People’s Hospital of Linxia, Linxia, Gansu Province China; 2 Department of Gynecology, The People’s Hospital of Linxia, Linxia, Gansu Province China

**Keywords:** Peritoneal dialysis-associated peritonitis, Peritoneal dialysate, Perceptibly hot dialysate-related peritoneal injury, Septic shock, Home peritoneal dialysis, Prevention and control of dialysis complications

## Abstract

**Background:**

Peritoneal dialysis-associated peritonitis mostly arises from fluid exchange contamination, catheter tunnel exit infection or intestinal flora translocation. Peritoneal thermal injury triggered by perceptibly hot peritoneal dialysate, which advances to secondary suppurative peritonitis and fatal septic shock, is extremely rare. Few published studies have documented complete clinical courses of this life-threatening complication. Such incidents are often overlooked in routine home peritoneal dialysis administration. Experimental evidence confirms dialysate temperatures exceeding 43 °C cause irreversible peritoneal barrier destruction, while the standard infusion temperature is 36–37 °C.

**Case presentation:**

A 57-year-old male on long-term home peritoneal dialysis suffered severe acute abdominal pain immediately after infusing perceptibly hot peritoneal dialysate caused by temperature control failure of his heating device. He rapidly developed purulent turbid dialysate, markedly elevated inflammatory markers and refractory septic shock. Multidisciplinary comprehensive interventions including broad-spectrum antibiotics, haemodynamic stabilisation and CRRT could not reverse his critical condition. The patient died one day after his family elected to discontinue active treatment. We report the full clinical data of this case and systematically analyse its potential pathogenesis, disease progression, key differential diagnoses and full-course therapeutic strategies.

**Conclusions:**

Heating equipment malfunction and inadequate standardised temperature management can lead to injury induced by perceptibly hot peritoneal dialysate, which initiates a sequential cascade of aseptic chemical inflammation and severe polymicrobial infection. Clinicians should strengthen standardised heating procedures and tiered patient education to prevent this rare yet highly fatal dialysis adverse event. This case delivers vital clinical alerts for temperature safety supervision in home peritoneal dialysis.

**Supplementary Information:**

The online version contains supplementary material available at https://doi.org/10.1186/s12882-026-05314-0.

## Introduction

Peritoneal dialysis represents a mainstream home-based renal replacement therapy for patients with end-stage kidney disease, characterised by stable haemodynamics and extensive clinical application [1, 2]. Peritoneal dialysis-associated peritonitis is a severe dialysis complication that markedly increases hospitalisation risks and impairs long-term dialysis outcomes [3, 4]. This complication is predominantly induced by fluid exchange contamination, catheter-related infection and intestinal bacterial translocation [5, 6].

Clinical guidelines recommend pre-warming peritoneal dialysate to 36–37 °C prior to infusion to match the physiological intraperitoneal environment. Existing studies have demonstrated that elevated dialysate temperatures damage peritoneal mesothelial cells; temperatures exceeding 43 °C lead to irreversible destruction of the peritoneal barrier and subsequent intraperitoneal inflammatory responses [7].

After searching CNKI, Wanfang and PubMed databases, we found that complete clinical reports describing acute peritoneal thermal injury complicated by severe suppurative peritonitis and septic shock following infusion of perceptibly hot dialysate are scarce worldwide. Only one case published by Lai et al. documented delayed intestinal perforation after administration of dialysate with abnormally elevated temperature [8]. Current research regarding peritoneal thermal injury mainly focuses on hyperthermic intraperitoneal chemotherapy for malignant tumors [9–11], while no systematic clinical summaries have addressed adverse events caused by temperature malfunctions in home peritoneal dialysis. Herein, we report a patient who developed fatal suspected peritoneal thermal injury combined with polymicrobial peritonitis after infusing perceptibly hot dialysate due to heating device failure. Combined with the full clinical timeline, laboratory results and published basic research data, we discuss its potential pathogenesis, differential diagnostic considerations and standardized prevention strategies, aiming to provide clinical references for temperature safety management in home peritoneal dialysis.

## Case presentation

A 57-year-old male taxi driver was admitted on Day 0 (the day of admission) with a chief complaint of sudden abdominal pain lasting 3 h after infusing perceptibly hot peritoneal dialysate, with a 5-year history of renal dysfunction and a 3-year history of peritoneal dialysis.

Five years prior to admission, the patient was diagnosed with hypertension at an external hospital, with a maximum blood pressure of 180/100 mmHg. Laboratory tests at our hospital revealed blood urea 15.1 mmol/L and serum creatinine 343.3 µmol/L, and he received conservative medical management. Four years ago, renal puncture biopsy was performed at the Second Hospital of Lanzhou University. Pathological findings indicated chronic tubulointerstitial nephritis with suspicious κ light chain crystalline casts within renal tubules. Three years ago, he was diagnosed with end-stage chronic kidney disease (uremia). After peritoneal dialysis catheter implantation, home peritoneal dialysis was initiated. His dialysis regimen consisted of 2000 mL low-calcium 1.5% peritoneal dialysate administered three times daily, plus 2000 mL low-calcium 2.5% peritoneal dialysate for overnight dwell. His daily ultrafiltration volume was approximately 1000 mL, and he was anuric. The patient had irregular outpatient follow-up and suffered from long-standing chronic wasting disease.

Three hours before admission, temperature control failure occurred in his home peritoneal dialysis heating device. According to the patient’s statement, the dialysate felt perceptibly hot when he took it out, yet he did not discard the fluid and infused the entire bag into his peritoneal cavity directly. Restricted by domestic conditions, the actual temperature of the dialysate was not measured at the scene, and objective temperature data can no longer be obtained. Abdominal pain developed immediately after infusion and worsened progressively after fluid exchange, presenting as persistent epigastric and periumbilical pain accompanied by nausea, vomiting of gastric contents, and yellow watery diarrhoea. The patient visited our emergency department immediately and was admitted with a tentative diagnosis of acute gastroenteritis, peritoneal dialysis status, and end-stage chronic kidney disease (uremia).

## Admission physical examination

Vital signs: temperature 37.0 °C, pulse 82 beats per minute, respiratory rate 20 breaths per minute, blood pressure 108/78 mmHg; height 163 cm, weight 55 kg. The patient was alert but lethargic with an acute illness appearance, and no jugular venous distension was observed. His heart rate was regular at 82 beats/min with no pathological murmurs; bilateral lung auscultation revealed clear breath sounds without crackles or rhonchi. The peritoneal dialysis catheter was well secured, with no erythema, exudate or purulent discharge at the catheter exit site. The abdomen was soft, with tenderness over the epigastrium and periumbilical area, without guarding or rebound tenderness, and bowel sounds were normal. Limb muscle strength and tone were intact, with mild bilateral lower-limb oedema.

## Laboratory and imaging findings

### Investigations on Day 0

Complete blood count (CBC): white blood cell (WBC) 12.04 × 10⁹/L, neutrophil percentage 91.00%, absolute neutrophil count 10.96 × 10⁹/L, red blood cell (RBC) 3.40 × 10¹²/L, haemoglobin 103 g/L, platelet 152 × 10⁹/L; C-reactive protein (CRP) 11.48 µg/mL.

Coagulation profile: prothrombin time (PT) 19.20 s, international normalised ratio (INR) 1.640, activated partial thromboplastin time (APTT) 46.40 s, fibrin degradation products (FDP) 6.00 µg/mL, D-dimer 1.58 µg/mL.

Serum amylase: 87 U/L.

N-terminal pro-BNP: 2244.6 pg/mL; serum troponin I: 0.03 ng/mL.

Serum biochemistry: albumin 19.0 g/L, serum creatinine 976.5 µmol/L, urea 22.40 mmol/L, carbon dioxide combining power 22.5 mmol/L, potassium 4.28 mmol/L, calcium 1.99 mmol/L, chloride 99.00 mmol/L, sodium 144.30 mmol/L.

Cardiac enzymes: creatine kinase (CK) 878 U/L, CK-MB mass concentration 24.730 ng/mL.

Initial peritoneal dialysate routine analysis: clear pale yellow fluid; WBC 30 × 10⁶/L (90% neutrophils, 10% lymphocytes), RBC 10 × 10⁶/L.

Stool examination: negative for occult blood, red and white blood cells.

Electrocardiogram and chest radiograph showed no remarkable abnormalities.

On Day 0, only routine biochemistry and cell count tests were performed on peritoneal dialysate. Physical examination revealed merely abdominal tenderness without rebound tenderness or muscular rigidity, and the dialysate remained clear without turbidity. Combined with dialysate laboratory results, a diagnosis of peritonitis lacked sufficient evidence, so bacterial culture of dialysate was not submitted at that time. Empiric intravenous ceftazidime 1.0 g twice daily plus antispasmodic symptomatic therapy was administered, yet the patient’s abdominal pain did not improve satisfactorily. His condition deteriorated rapidly the next day and progressed to septic shock, which required upgraded broad-spectrum antimicrobial therapy; detailed therapeutic measures are described in the Day 1 subsection below.

### Clinical deterioration and examinations on Day 1 (2nd hospital day)

**Fig. 1:**
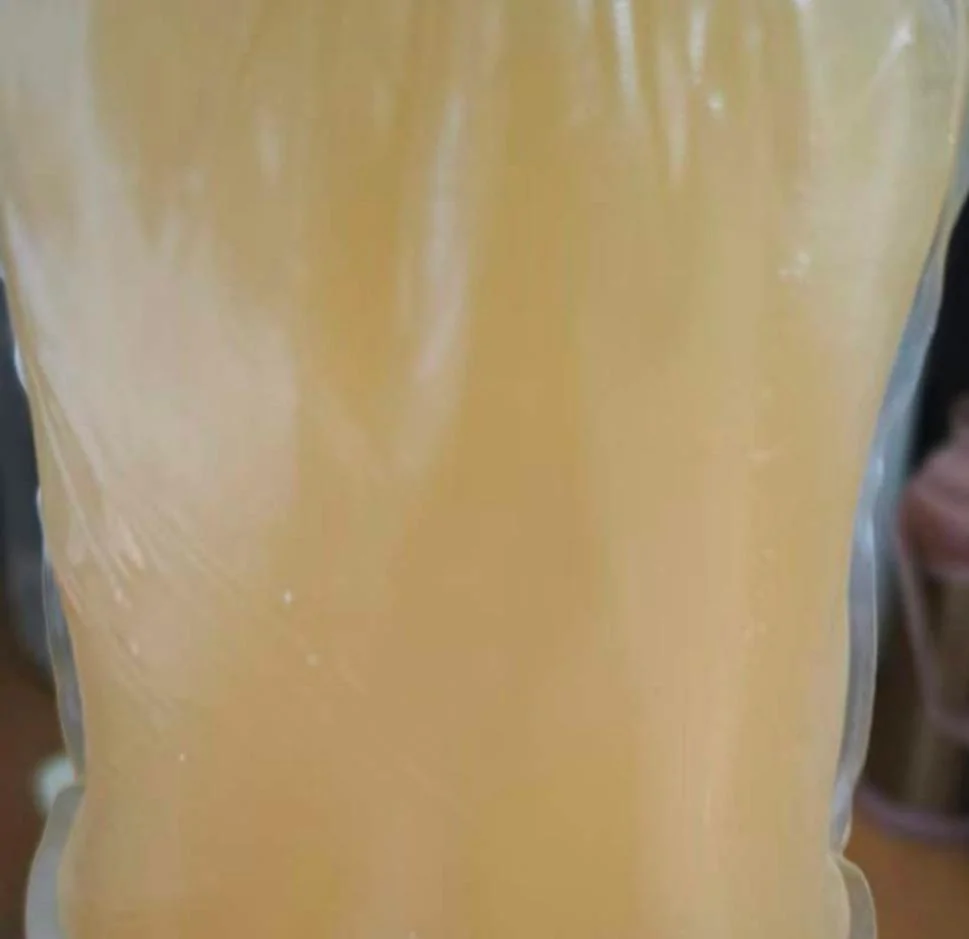
Purulent-turbid peritoneal dialysate sampled on Day 1 (hospital-admission Day 2)

The patient suffered abrupt clinical deterioration with fever (peak temperature 39.2 °C), hypotension (blood pressure 85/40 mmHg), tachycardia (heart rate 119 beats/min) and tachypnoea (respiratory rate 30 breaths/min). Abdominal tenderness worsened with new-onset rebound tenderness and markedly diminished bowel sounds, showing typical signs of peritoneal irritation. His peritoneal dialysate turned yellow and purulent-turbid (Fig. 1). Peripheral venous blood and peritoneal dialysate samples were collected simultaneously for microbial culture. Repeat inflammatory markers were markedly elevated: procalcitonin (PCT) 52.910 ng/mL, interleukin-6 (IL-6) 2445.59 pg/mL.

Repeat CBC: WBC 18.51 × 10⁹/L, neutrophil percentage 86.30%, absolute neutrophil count 15.97 × 10⁹/L, CRP 107.26 µg/mL.

Peritoneal dialysate analysis: WBC 800 × 10⁶/L (90% neutrophils, 10% lymphocytes), RBC 90 × 10⁶/L.

Arterial blood gas: pH 7.41, partial pressure of carbon dioxide (PaCO₂) 28.5 mmHg, partial pressure of oxygen (PaO₂) 79.0 mmHg, lactate 3.0 mmol/L, bicarbonate 18.0 mmol/L, consistent with metabolic acidosis complicated by respiratory alkalosis.

**Fig. 2:**
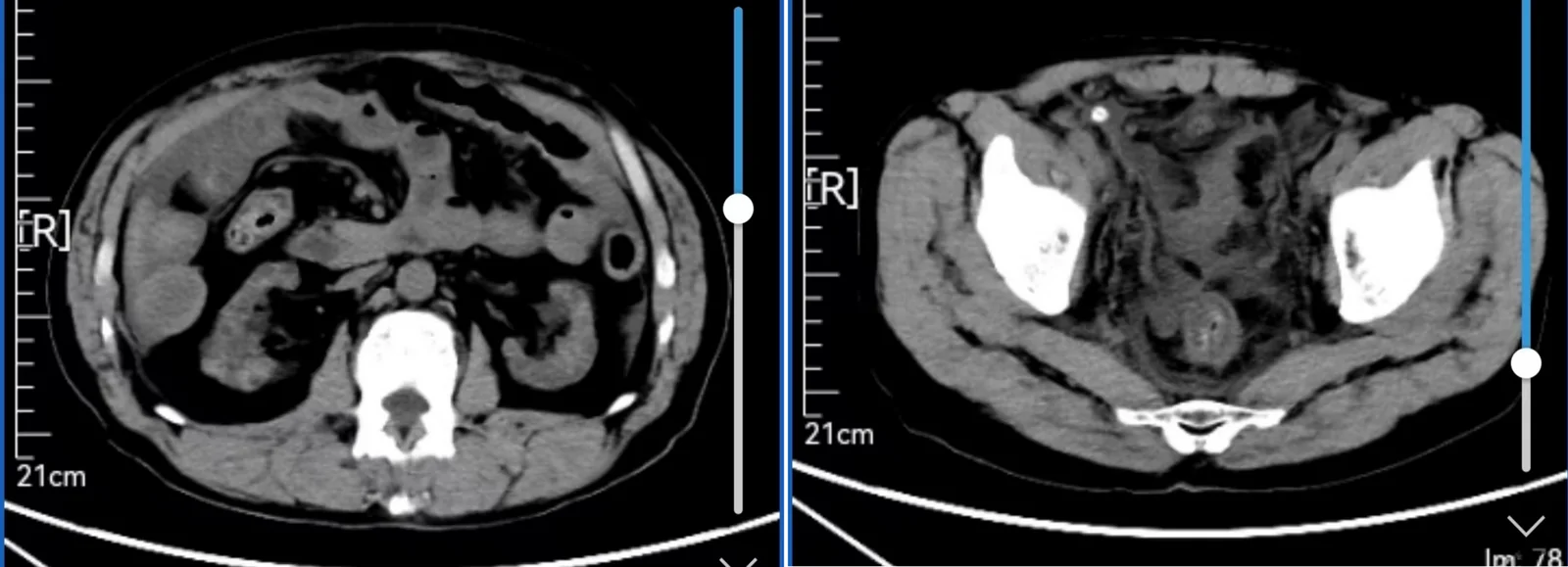
Plain abdominopelvic CT acquired on Day 1, demonstrating diffuse intestinal-wall thickening and intra-abdominal inflammatory exudate

Plain abdominopelvic CT scan demonstrated peritonitis, diffuse intestinal wall thickening and inflammatory infiltration, suggestive of incomplete intestinal obstruction, together with diffuse bilateral renal lesions and bilateral renal atrophy (Fig. 2).

Echocardiography showed left atrial enlargement, aortic sclerosis, grade I left ventricular diastolic dysfunction with preserved left ventricular systolic function (left ventricular ejection fraction, LVEF 65%), and mild grade I aortic regurgitation.

Combined with clinical manifestations, physical signs and auxiliary examinations, revised diagnoses were established: septic shock, sepsis, peritoneal dialysis-associated peritonitis, incomplete intestinal obstruction, end-stage chronic kidney disease (uremia), peritoneal dialysis-dependent status. A multidisciplinary consultation involving nephrology, general surgery, intensive care unit (ICU), gastroenterology and clinical pharmacy was held immediately to formulate a treatment regimen: nil per os with continuous gastrointestinal decompression (daily gastric drainage volume 800–1700 mL) and fluid resuscitation; metaraminol was administered by continuous intravenous pump infusion at a rate of 5 mg/h [1.52 µg/(kg·min)] from Day 1 to Day 10. The infusion speed was dynamically titrated according to real-time blood pressure to maintain haemodynamic stability. The antimicrobial regimen was adjusted to intravenous imipenem-cilastatin sodium 0.5 g twice daily combined with intraperitoneal instillation 0.25 g twice daily. Adjunctive interventions included antispasmodic treatment, intraperitoneal lavage via the dialysis catheter, nutritional support, and human albumin infusion to correct hypoalbuminaemia. A temporary right internal jugular venous catheter was placed for bedside continuous renal replacement therapy (CRRT) to stabilise internal homeostasis.

### Clinical course: Day 2 to Day 10

#### Day 2–Day 6 (3rd–7th hospital days)

The patient remained critically ill with intermittent fever. His abdominal pain failed to achieve substantial relief, and peritoneal dialysate stayed purulent and turbid with persistent peritoneal irritation signs. Vasopressors were still required to sustain blood pressure, and the comprehensive treatment regimen described above was continued without modification.

#### Day 7 (8th hospital day)

**Fig. 3:**
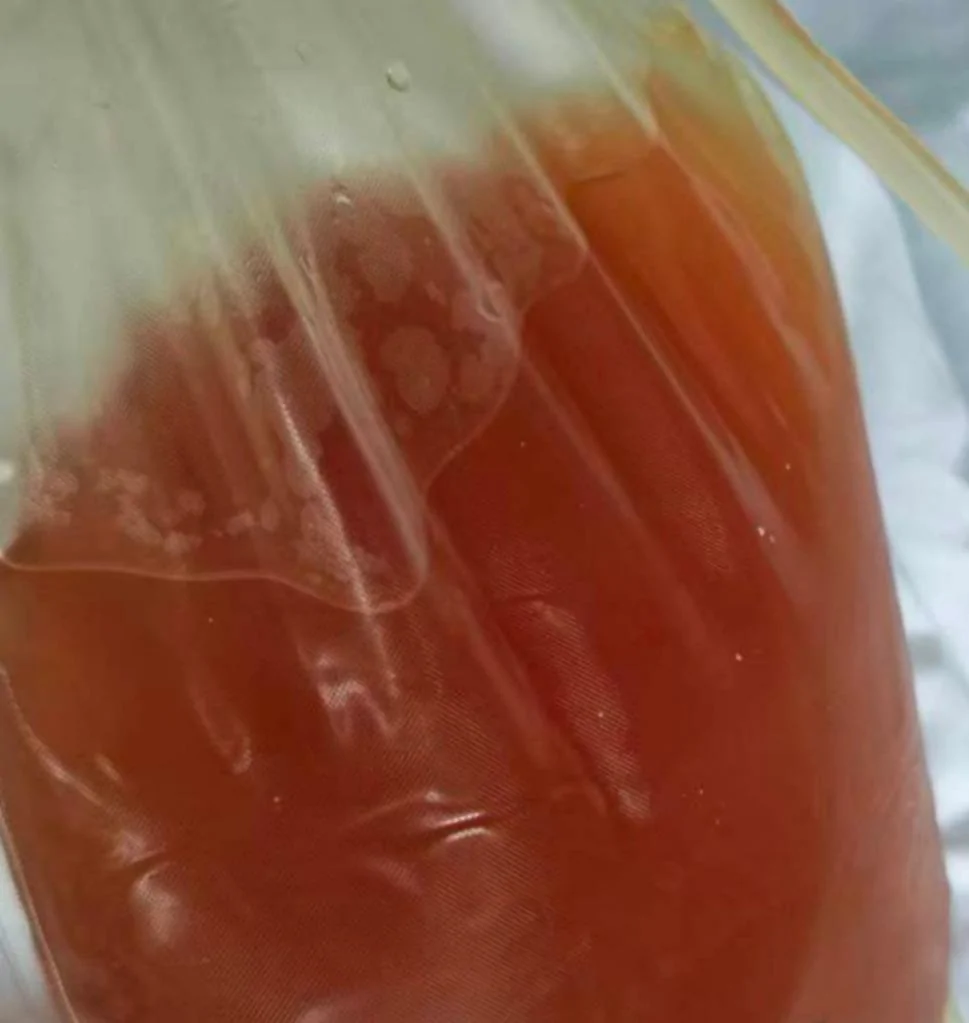
Haemorrhagic peritoneal dialysate sampled on Day 7 (hospital-admission Day 8)

Gastric drainage fluid turned pale red with weakly positive occult blood test results; the peritoneal dialysate also appeared bloody (Fig. 3). Proton pump inhibitors and gastric mucosal protective agents were immediately added for symptomatic management, while all other therapies remained unchanged.

After one week of standardized comprehensive treatment, serial inflammatory markers in peripheral blood and peritoneal dialysate showed no declining trends (Table 1, provided before the reference section at the end of manuscript), and the patient’s clinical status failed to improve. Based on bacterial culture results from peripheral blood, peritoneal dialysate and stool specimens (Table 2, provided before the reference section at the end of manuscript), intravenous linezolid 600 mg twice daily was added to the existing intravenous imipenem-cilastatin regimen for combined antibacterial therapy, with intraperitoneal antimicrobial instillation unchanged.

**Table 1:** Changes in inflammatory biomarkers in peripheral blood and peritoneal dialysate

Specimen Type	Test Item	Day 0	Day 1	Day 3	Day 4	Day 5	Day 7	Day 8	Day 10
Peripheral blood	WBC (×10⁹/L)	12.04	18.51	6.26	Not performed	10.17	19.26	29.79	28.18
	NEUT%	91.00%	86.30%	88.70%	Not performed	86.40%	85.90%	94.50%	88.80%
	NEUT# (×10⁹/L)	10.96	15.97	5.54	Not performed	8.79	16.54	28.15	25.02
	CRP (µg/mL)	11.48	107.26	173.75	Not performed	163.52	130.79	196.87	168.95
	PCT (ng/mL)	Not performed	52.91	Not performed	Not performed	> 100	74.53	Not performed	28.27
Peritoneal dialysate	WBC (×10⁶/L)	30	800	Not performed	1260	Not performed	Not performed	Not performed	3190
	N%	90.00%	90.00%	Not performed	90.00%	Not performed	Not performed	Not performed	80.00%
	RBC (×10⁶/L)	10	90	Not performed	360	Not performed	Not performed	Not performed	3570

Notes: WBC, white blood cell count; NEUT%, peripheral blood neutrophil percentage; NEUT#, absolute neutrophil count; CRP, C-reactive protein; PCT, procalcitonin; RBC, red blood cell count; N%, neutrophil percentage in peritoneal dialysate. Blank entries denote that relevant laboratory tests were not performed on the corresponding day

**Table 2:** Results of pathogen culture from peripheral blood, peritoneal dialysate and stool

Sampling date (Report date)	Peritoneal dialysate culture	Blood culture	Stool culture
Day 1(Report: Day 8)	*Escherichia coli* (MDR strain) *Enterococcus faecium* (Susceptible to linezolid and vancomycin)	No bacterial growth	Not performed
Day 4(Report: Day 9)	*Escherichia coli* (MDR strain)	Not performed	Not performed
Day 4(Report: Day 12)	*Enterococcus faecalis* (Susceptible to linezolid and vancomycin)	Not performed	Not performed
Day 7(Report: Day 13)	Not performed	No bacterial growth	Not performed
Day 8(Report: Day 12)	*Enterococcus faecium* (Susceptible to linezolid and vancomycin) *Enterobacter cloacae*	Not performed	Not performed
Day 9(Report: Day 12)	Not performed	Not performed	No pathogenic bacteria isolated

Notes: MDR, multidrug-resistant; Not performed: specimens were not submitted for culture on the corresponding date; No bacterial growth: no cultivable bacteria were isolated from samples; No pathogenic bacteria isolated: no clinically significant pathogens were detected in stool specimens

#### Day 8–Day 9 (9th–10th hospital days)

**Fig. 4:**
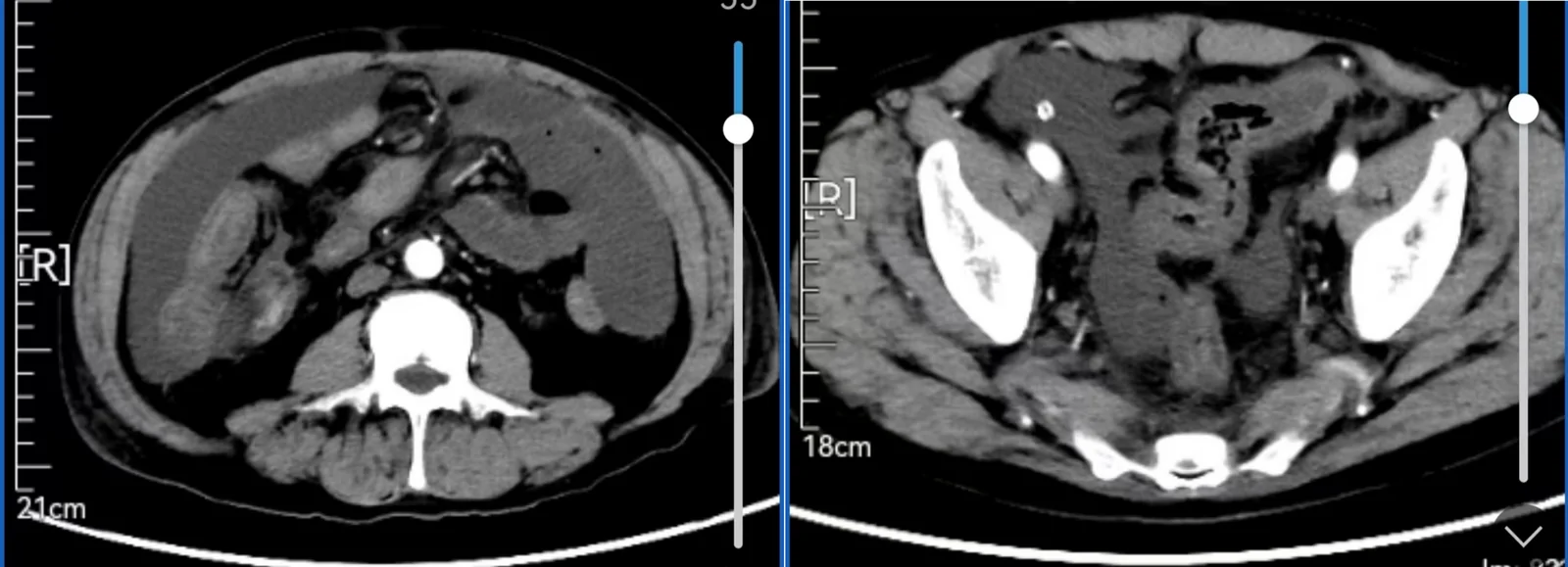
Contrast-enhanced abdominopelvic CT acquired on Day 8 showing intestinal-wall oedema secondary to peritonitis, with no evidence of intestinal ischaemia or gastrointestinal perforation

Recurrent high-grade fever persisted, and vasopressor support remained mandatory for blood pressure maintenance. Abdominal tenderness and rebound tenderness persisted, with absent bowel sounds, indicating critical overall condition. Contrast-enhanced abdominopelvic CT was performed on Day 8 to exclude ischaemic necrotising enteritis and gastrointestinal perforation. The imaging results were as follows:

- Pelviabdominal effusion and peritonitis;
- Segmental intestinal wall thickening and oedema, predominantly involving the ascending colon;
- Suspected incomplete intestinal obstruction (Fig. 4).

No radiological hallmarks of free intraperitoneal gas, intestinal rupture or extensive intestinal ischaemic necrosis were visualised on this contrast CT. Acute gastrointestinal perforation and ischaemic necrotising enteritis were therefore not supported at this stage, although the latent risk of delayed intestinal perforation in the later clinical course could not be completely excluded. Detailed differential reasoning is elaborated in the Discussion section.

The patient’s condition deteriorated progressively throughout hospitalisation, with haemodynamics fully dependent on vasoactive agents. Integrating all clinical data, we highly suspect that the peritonitis in this case originated from peritoneal barrier injury triggered by infusion of perceptibly hot peritoneal dialysate, followed by intestinal bacterial translocation. The peritoneal dialysis catheter only acted as a secondary contributing factor; meanwhile, the catheter served as an effective channel to drain and lavage purulent intraperitoneal fluid to alleviate peritoneal inflammation. Given the patient’s labile vital signs, premature catheter removal carried high traumatic risks with minimal clinical benefit. We therefore retained the dialysis catheter temporarily and prioritised conservative management, including broad-spectrum antibiotics, nil per os, gastrointestinal decompression, metaraminol infusion, intraperitoneal lavage, CRRT and multi-organ supportive care. This risk-benefit balanced conservative strategy represents one limitation of clinical management in this critically ill patient.

#### Day 10 (11th hospital day)

The patient’s clinical status continued to worsen. After full disclosure of critical illness and poor prognosis to his family, they elected to withdraw all active treatment and signed informed consent for self-discharge. Follow-up contact confirmed that the patient passed away one day after discharge.

The full chronological clinical course of the patient is summarized in Table 3 (provided before the reference section at the end of manuscript).

**Table 3:** Timeline of clinical manifestations and corresponding interventions

Timepoint	Core clinical manifestations	Imaging examinations	Laboratory & microbiological findings	Therapeutic interventions
Day 0 (admission day)	Admitted with 3 h of sudden abdominal pain after infusion of perceptibly hot peritoneal dialysate. Vital signs: T 37 °C, pulse 82 beats/min, RR 20 breaths/min, BP 108/78 mmHg. The patient appeared acutely ill and lethargic, with epigastric and periumbilical tenderness; no guarding or rebound tenderness, and normal bowel sounds.	Electrocardiogram and chest radiograph revealed no abnormalities.	CBC: WBC 12.04 × 10⁹/L, NEUT% 91.00%, CRP 11.48 µg/mL; serum creatinine 976.5 µmol/L, albumin 19.0 g/L; normal serum amylase; negative stool occult blood. Peritoneal dialysate analysis: clear pale yellow fluid, WBC 30 × 10⁶/L (90% neutrophils).	Tentative diagnosis of acute gastroenteritis. Empiric intravenous ceftazidime 1.0 g twice daily plus antispasmodic treatment. Abdominal pain failed to improve and the disease progressed continuously.
Day 1 (2nd hospital day)	Rapid clinical deterioration; fever with peak temperature 39.2 °C, hypotension (BP 85/40 mmHg), heart rate 119 beats/min. Positive peritoneal irritation signs and diminished bowel sounds; peritoneal dialysate turned yellow, purulent and turbid (Fig. 1).	Plain abdominopelvic CT: peritonitis, intestinal wall thickening and oedema suggestive of incomplete intestinal obstruction, bilateral renal atrophy (Fig. 2). Echocardiography: left atrial enlargement, grade I left ventricular diastolic dysfunction.	CBC: WBC 18.51 × 10⁹/L, NEUT% 86.30%, CRP 107.26 µg/mL; PCT 52.91 ng/mL, IL-6 2445.59 pg/mL; dialysate WBC 800 × 10⁶/L (90% neutrophils). Blood gas analysis indicated metabolic acidosis complicated by respiratory alkalosis. Peripheral blood and dialysate samples were collected for bacterial culture and antimicrobial susceptibility testing.	Revised diagnoses: septic shock, peritoneal dialysis-associated peritonitis. Multidisciplinary rescue initiated: nil per os, continuous gastrointestinal decompression. metaraminol infused continuously at 5 mg/h [1.52 µg/(kg·min)] (administered Day 1–Day 10, rate titrated dynamically based on real-time BP to stabilise circulation). Antibiotics upgraded to intravenous imipenem-cilastatin sodium 0.5 g twice daily combined with intraperitoneal instillation 0.25 g twice daily. Temporary venous catheter placed for bedside CRRT, alongside nutritional support, hypoalbuminaemia correction and intraperitoneal lavage via dialysis catheter.
Day 2–3 (3rd–4th hospital days)	Persistent high fever, purulent turbid dialysate, sustained peritoneal irritation signs; vasopressors still required for blood pressure support.	No additional imaging performed.	CBC: WBC 6.26 × 10⁹/L, NEUT% 88.70%, CRP 173.75 µg/mL.	Maintenance of nil per os, gastrointestinal decompression, metaraminol infusion at Day 1 dosage, imipenem-cilastatin sodium, intraperitoneal lavage, CRRT and multi-organ supportive care.
Day 4–6 (5th–7th hospital days)	Unrelieved abdominal pain, recurrent fever, unstable vital signs dependent on vasopressors.	No additional imaging performed.	CBC: WBC 10.17 × 10⁹/L, NEUT% 86.40%, CRP 163.52 µg/mL; PCT > 100 ng/mL. Dialysate WBC rose to 1260 × 10⁶/L (90% neutrophils) with no downward trend in inflammatory markers. Repeat dialysate samples submitted for bacterial culture.	Original therapeutic regimen maintained with close monitoring of clinical status.
Day 7 (8th hospital day)	Pale red gastric drainage fluid; bloody peritoneal dialysate. Recurrent high fever, positive peritoneal irritation signs; vasopressor dependence persisted.	No additional imaging performed.	CBC: WBC 19.26 × 10⁹/L, NEUT% 85.90%, CRP 130.79 µg/mL; PCT 74.53 ng/mL; weakly positive occult blood in gastric aspirate.	Proton pump inhibitors and gastric mucosal protectants added for symptomatic management; all other prior therapies continued unchanged.
Day 8 (9th hospital day)	Critical condition with refractory abdominal pain and positive peritoneal irritation signs; vasopressors still required.	Contrast-enhanced abdominopelvic CT: pelviabdominal effusion, peritonitis, ascending colon wall thickening and oedema with suspected incomplete intestinal obstruction (Fig. 4). No radiological signs of pneumatosis intestinalis, extensive intestinal necrosis or free intraperitoneal gas to suggest intestinal ischaemia or gastrointestinal perforation, though delayed intestinal perforation risk could not be fully excluded.	Initial culture results: multidrug-resistant (MDR) *Escherichia coli *and *Enterococcus faecium* isolated from dialysate (susceptible to linezolid and vancomycin); peripheral blood cultures yielded no bacterial growth (Table 2).	Imaging ruled out acute intestinal ischaemic necrosis and gastrointestinal perforation. Intravenous linezolid 600 mg twice daily added for combined anti-infection therapy. Nil per os, gastrointestinal decompression, metaraminol infusion, intraperitoneal lavage, CRRT and organ support continued. Haemodynamics remained severely unstable throughout admission; catheter removal carried high trauma risk with limited clinical benefit, so the peritoneal dialysis catheter was retained for conservative supportive care and purulent fluid lavage.
Day 9 (10th hospital day)	Persistently critical condition, unrelieved abdominal pain, positive peritoneal irritation signs, ongoing vasopressor requirement.	No additional imaging performed.	Repeat cultures: MDR *E. coli* and *Enterococcus faecalis* isolated from dialysate (susceptible to linezolid and vancomycin) (Table 2).	Continuous nil per os, gastrointestinal decompression, metaraminol infusion, dual antibiotics (imipenem-cilastatin sodium at Day 1 dosage plus linezolid at Day 8 dosage), intraperitoneal lavage, CRRT and multi-organ support. Catheter retention rationale consistent with Day 8.
Day 10 (11th hospital day)	Continuous high fever, intractable abdominal pain, positive peritoneal irritation signs; vasopressor support still necessary.	No additional imaging performed.	CBC: WBC 28.18 × 10⁹/L, CRP 168.95 µg/mL, PCT 28.27 ng/mL; dialysate WBC climbed to 3190 × 10⁶/L.	The patient’s family received full disclosure of critical illness and poor prognosis and elected to discontinue all active treatment. The patient was discharged against medical advice after signing informed consent, and follow-up confirmed death 1 day post-discharge.

Notes: WBC, white blood cell count; NEUT%, neutrophil percentage; CRP, C-reactive protein; PCT, procalcitonin; IL-6, interleukin-6; MDR, multidrug-resistant; CRRT, continuous renal replacement therapy

## Discussion

### Plausible stepwise pathogenesis of peritoneal injury induced by perceptibly hot peritoneal dialysate

The physiological intraperitoneal temperature of human bodies remains stably at 36–37 °C. This temperature range preserves intact peritoneal mesothelial cell architecture, stable peritoneal barrier function and local intraperitoneal immunity, and serves as the recommended infusion temperature for clinical peritoneal dialysate. Published studies have demonstrated that peritoneal dialysate temperatures exceeding the safe threshold trigger hierarchical, progressive peritoneal injury, which follows a three-stage pathological sequence: primary thermal damage, secondary chemical inflammation, and bacterial infection [9, 12]. We hypothesise that this full injury cascade was triggered by perceptibly hot peritoneal dialysate in our patient.

Infusion of perceptibly hot dialysate is considered the suspected initiating trigger of clinical deterioration in this case. Basic experimental evidence shows that dialysate at 41–42 °C causes mesothelial cell oedema and loss of surface microvilli, representing mild, reversible injury. Temperatures ≥ 43 °C lead to widespread mesothelial necrosis and exfoliation with exposed basement membranes, resulting in irreversible disruption of the peritoneal barrier [9]. At temperatures ≥ 45 °C, peritoneal tissue undergoes thermal coagulative necrosis, microvascular occlusion and interstitial haemorrhage accompanied by heavy inflammatory cellular infiltration [10]. This completely disrupts intestinal mechanical and mucosal immune barriers, impairs immune function of intestinal epithelium and mesenteric lymphoid tissue, and consequently facilitates bacterial translocation [7, 13, 14]. In our patient, the heating device suffered temperature control malfunction, and the dialysate felt distinctly hot upon removal, clearly exceeding the tolerable perceptual temperature for infusion. Combining documented thermal injury patterns and the patient’s subsequent severe peritoneal and intestinal mucosal lesions, we infer the infused dialysate most likely reached ≥ 45 °C, acting as the inciting factor for peritoneal injury and subsequent septic shock.

Following peritoneal thermal injury, necrotic mesothelial cells release abundant pro-inflammatory mediators including tumour necrosis factor and interleukins, activating key inflammatory signalling pathways such as NF-κB and inducing severe intraperitoneal aseptic chemical inflammation. This manifests as diffuse peritoneal congestion and oedema, abnormally elevated vascular permeability and increased intraperitoneal exudation, with persistent severe abdominal pain and tenderness as core peritoneal irritation signs [9]. Once peritoneal barrier integrity is compromised, local intraperitoneal immune defence declines markedly, allowing enteric opportunistic bacteria to translocate, colonise and multiply massively. The initial aseptic inflammation then progresses to suppurative bacterial peritonitis, characterised by purulent turbid dialysate and sharply elevated inflammatory markers, which ultimately culminates in septic shock [8]. In this patient, severe abdominal pain developed immediately after infusion of perceptibly hot peritoneal dialysate, followed sequentially by purulent turbid dialysate, bloody dialysate, uncontrolled systemic inflammatory response and eventual progression to septic shock. Based on the above pathological mechanisms, we hypothesise that perceptibly hot peritoneal dialysate triggered a complete cascade: peritoneal injury → intraperitoneal aseptic inflammation → intestinal bacterial translocation.

**Fig. 5:**
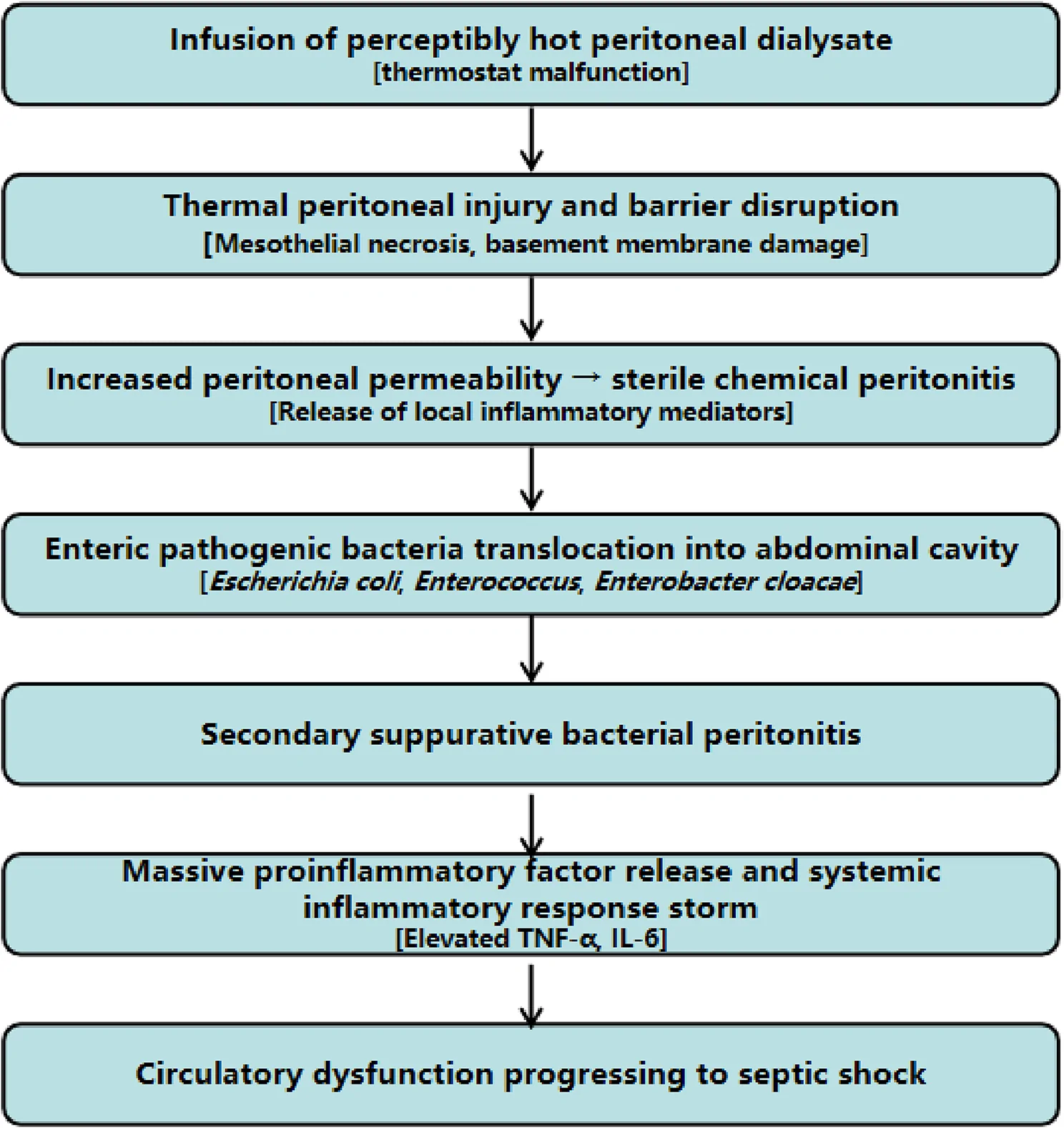
Hypothesised pathogenic cascade of sepsis induced by perceptibly hot peritoneal dialysate in this patient

The progressive injury cascade summarised in this article is as follows: thermal stimulation from perceptibly hot peritoneal dialysate → peritoneal injury → peritoneal barrier breakdown → aseptic chemical inflammation → translocation of enteric pathogenic bacteria → polymicrobial suppurative peritonitis → systemic inflammatory response storm → septic shock (Fig. 5). This cascade is only a hypothesis derived from this single patient. Restricted to limited individual clinical data, with no measured dialysate temperature or peritoneal histopathological specimens available, this sequential pathological pathway cannot be definitively verified as an objective disease process. Furthermore, the patient had long-standing severe hypoalbuminaemia, which impaired peritoneal self-repair capacity and local intraperitoneal immune defence. This underlying condition amplified the inflammatory cascade after peritoneal injury and accelerated progression to septic shock, representing an individualised high-risk factor that accounted for the fulminant clinical course.

### Rarity of the case and differential diagnostic analysis

To our knowledge, no systematic clinical studies have been published globally documenting the full clinical course following infusion of perceptibly hot peritoneal dialysate complicated by severe suppurative peritonitis, septic shock and death. Only one single case report has described delayed intestinal perforation secondary to abnormally elevated dialysate temperatures. Such adverse events are extremely rare in home peritoneal dialysis and carry high mortality rates.

Under standard heating protocols, peritoneal dialysate is maintained at the recommended 36–37 °C. Brief exposure to mildly heated fluid (mostly below 40 °C) typically only causes transient abdominal discomfort, without irreversible peritoneal barrier injury or subsequent severe intraperitoneal infection. This explains the scarcity of systematically reported complete clinical cases of this complication.

In this patient, perceptibly hot dialysate was infused despite temperature control malfunction of the heating device. Compound by long-standing severe hypoalbuminaemia that impaired peritoneal repair capacity and local intraperitoneal immunity, the patient rapidly progressed to fatal septic shock. This case presents a complete clinical cascade with typical manifestations, delivering strong clinical warnings for temperature safety regulation in home peritoneal dialysis.

Four differential diagnoses are outlined to avoid clinical misdiagnosis:

1. Chemical peritonitis: Mainly induced by unbalanced dialysate osmolarity, abnormal pH or residual catheter disinfectants, without exposure to perceptibly hot peritoneal dialysate infusion [15, 16].
2. Conventional bacterial peritonitis: Triggered by contamination during fluid exchange, catheter tunnel infection or spontaneous intestinal flora translocation; abdominal pain onset bears no immediate temporal correlation with dialysate infusion [5, 6].
3. Ischaemic necrotising enteritis (core differential diagnosis): Caused by impaired intestinal microcirculatory perfusion, with no dialysis-related acute trigger. Classic manifestations include haematochezia and persistent paralytic ileus, alongside characteristic CT findings of pneumatosis intestinalis and intestinal necrosis [17]. Our patient had an immediate trigger of hot dialysate infusion. Pale gastric drainage fluid with weakly positive occult blood only indicated stress injury of the gastrointestinal mucosa, without massive haematemesis or persistent melena. Contrast-enhanced CT revealed no signs of intestinal ischaemia or necrosis, so this diagnosis was excluded.
4. Gastrointestinal perforation (key differential diagnosis): Mostly secondary to intestinal ulceration or intestinal ischaemic injury. The hallmark radiological feature is free intraperitoneal gas, accompanied clinically by melena, haematemesis, faecal ascites and intractable severe vomiting [18]. Two abdominal CT scans obtained on Day 1 and Day 8 detected no free intraperitoneal gas or intestinal rupture, and no faecal debris was observed in peritoneal effluent. Acute gastrointestinal perforation could be ruled out at this stage, yet the latent risk of delayed intestinal perforation during later hospitalisation could not be fully eliminated.

The patient developed immediate abdominal pain after infusion of perceptibly hot peritoneal dialysate, accompanied by vomiting throughout admission. Continuous gastrointestinal decompression yielded 800–1700 mL gastric fluid daily. Bloody peritoneal dialysate and pale red gastric drainage with positive occult blood emerged on Day 7. Abdominal CT scans on Day 1 and Day 8 both indicated incomplete intestinal obstruction. These clinical signs indirectly indicate concurrent severe injury to both the peritoneum and intestinal mucosa after hot dialysate infusion, with suspected full-thickness intestinal involvement. Bloody gastric effluent resulted from retention of gastric and intestinal fluid secondary to incomplete obstruction. The patient never suffered haematemesis or massive melena, and serial CT scans showed no radiological evidence of intestinal ischaemia or perforation, ruling out primary organic intestinal lesions. Based on the chronological sequence of disease onset, we infer that thermal stimulation from perceptibly hot dialysate damaged the peritoneal barrier, triggering intraperitoneal inflammation and stress injury of the gastrointestinal mucosa, which further advanced to severe intraperitoneal infection.

### Standardised temperature management and clinical prevention implications for home peritoneal dialysis

Combining this fatal case with published basic experiments and clinical literature regarding peritoneal thermal injury, we hypothesise that temperature control malfunction of home dialysis heaters and inadequate standardised regulation of dialysate temperatures constitute the core precipitating factors for this adverse event. This case delivers a critical safety alert for the administration of home peritoneal dialysis. Current patient education programmes predominantly focus on sterile fluid exchange and catheter care, while dedicated training covering dialysate heating standards and temperature safety remains insufficient, forming an easily overlooked safety blind spot in home dialysis management.

Unified clinical standards are specified as follows: the safe infusion temperature for peritoneal dialysate is 36–37 °C; temperatures exceeding 43 °C cause irreversible peritoneal injury. Any heating device with temperature defects must be withdrawn from use immediately. Unregulated heating methods including hot water immersion and microwave heating, as well as temperature estimation by hand touch, are strictly prohibited. Medical thermostats equipped with temperature lock functions are mandatory. Patients are required to inspect device status prior to each infusion and follow standardised heating protocols rigorously.

Our hospital and local home dialysis centres have implemented uniform heating regulations: only medical heaters with a factory-set maximum temperature limit of 38 °C are permitted, and all simple household heating devices are banned. All patients and caregivers must complete specialised heating training before conducting home dialysis independently. In response to this fatal adverse event, we have optimised our prevention strategies: one-on-one refresher training is provided for patients with a history of heater malfunctions, heating workflows are verified on-site during home visits, and warning labels regarding peritoneal thermal injury are posted to prevent identical incidents at source.

Stratified health education should be delivered to patients and their caregivers to clarify the dual hazards posed by perceptibly hot peritoneal dialysate. Short-term complications include severe abdominal pain, peritoneal barrier disruption, polymicrobial peritonitis and septic shock. Repeated subclinical thermal injury over the long term leads to peritoneal fibrosis and ultrafiltration failure, which compromise long-term dialysis outcomes. For high-risk groups with poor follow-up adherence, malnutrition, hypoalbuminaemia, anaemia or impaired immunity, follow-up frequency should be increased, and underlying comorbidities actively corrected to improve peritoneal regenerative capacity and systemic immunity, thus reducing the risk of severe secondary infection after thermal injury.

Clinicians evaluating dialysis patients presenting with immediate abdominal pain following fluid exchange must first enquire about heater function, heating methods and dialysate temperature to identify suspected peritoneal thermal injury at an early stage and deliver timely intervention. This prevents mild conditions from progressing to fatal sepsis and safeguards the overall safety of patients receiving home peritoneal dialysis.

## Conclusion

Heater malfunction and non-standard heating operations can generate perceptibly hot peritoneal dialysate, which triggers stepwise damage to the peritoneal barrier. The clinical condition evolves from aseptic chemical inflammation to suppurative peritonitis, and may further progress to septic shock in critically ill patients. This dialysis complication mediated by suspected peritoneal thermal injury is rare, rapidly progressive and highly lethal, yet it can be effectively prevented via standardised dialysate temperature control and regulated heating procedures. To date, relevant case reports at home and abroad remain scarce, and this hazard has not attracted adequate clinical attention.

In addition, no dialysate samples or real-time temperature records were preserved at the time of the incident; no intraoperative peritoneal exploration or peritoneal histopathological biopsy was performed, and autopsy was unavailable after the patient’s death. Hence, the actual extent of peritoneal and intestinal injury cannot be directly confirmed by pathological evidence. The pathogenic pathway proposed herein — in which perceptibly hot peritoneal dialysate induces peritoneal thermal injury followed by severe infection and septic shock — is merely a reasonable clinical inference drawn from the patient’s full disease course, clinical signs and imaging findings. The progressive injury cascade summarised in this study is a case-specific hypothesis without universal applicability and cannot be generalised to all patients on home peritoneal dialysis. Clinicians should further optimise the safety management system for home dialysis heating equipment, unify standard heating workflows and routine equipment inspection protocols, implement stratified health education programmes, and rigorously regulate dialysate infusion temperatures to reduce the incidence of severe complications and improve long-term outcomes for peritoneal dialysis patients.

## Supplementary Information

Below is the link to the electronic supplementary material.

Supplementary Material 1

## Data Availability

All raw clinical data supporting the findings of this case report are securely preserved in the electronic medical record system of Linxia Prefectural People’s Hospital, Gansu Province. Relevant anonymized clinical data can be obtained from the corresponding author upon reasonable formal request.

## Abbreviations

BP: Blood pressure
RR: Respiratory rate
WBC: White blood cell count
NEUT%: Peripheral blood neutrophil percentage
NEUT#: Absolute neutrophil count
RBC: Red blood cell count
N%: Neutrophil percentage in peritoneal dialysate
CRP: C-reactive protein
PCT: Procalcitonin
MDR: Multidrug-resistant
CRRT: Continuous renal replacement therapy
NF-κB: Nuclear factor-kappa B
