# Supplementary material for: A case of suspected peritoneal thermal injury complicated by septic shock following infusion of perceptibly hot peritoneal dialysate
Source: BMC Nephrol. 2026 Aug 31;27:677. doi: 10.1186/s12882-026-05314-0 (PMC13769159; doi:10.1186/s12882-026-05314-0)
Supplement: Supplementary file 1 [file 12882_2026_5314_MOESM1_ESM.docx]

# Supplementary Table 1 Key Clinical Lessons and Standardized Preventive Strategies for Peritoneal Thermal Injury in Home Peritoneal Dialysis

| **Category** | **Detailed content** |
| --- | --- |
| 1. **Core Clinical Lessons** | 1. Infusion of perceptibly hot peritoneal dialysate may trigger peritonitis and even septic shock, representing a rare yet life-threatening adverse event in home peritoneal dialysis. Malfunction of warming devices and inadequate temperature control are highly suspected triggers in this case, serving as a critical safety warning for home dialysis management.  2. Published clinical guidelines specify the safe infusion temperature of peritoneal dialysate as 36–37 °C. Temperatures exceeding 43 °C may lead to irreversible damage to the peritoneal barrier.  3. The proposed pathogenic cascade of peritoneal thermal injury: perceptibly hot peritoneal dialysate damages the peritoneal barrier, subsequently inducing suppurative peritonitis and fatal septic shock. Repeated subclinical thermal stimulation may cause peritoneal fibrosis and ultrafiltration failure, compromising long-term peritoneal dialysis prognosis.  4. Current home dialysis education mainly focuses on aseptic exchange techniques, while risks associated with dialysate warming and temperature regulation are frequently overlooked, forming a neglected safety blind spot.  5. For peritoneal dialysis patients presenting with acute abdominal pain immediately after dialysate exchange, clinicians should first enquire about heater function and warming habits to identify suspected peritoneal thermal injury at an early stage and initiate timely intervention, preventing progression to severe systemic infection and septic shock. |
| **2.Standardized Preventive Measures for Home Peritoneal Dialysis** | 1. Unified standards for equipment and operationImplement standardized temperature control for peritoneal dialysate, with a safe infusion range of 36–37 °C; temperatures above 43 °C may result in irreversible peritoneal injury.Any warming device with temperature malfunctions must be withdrawn from use immediately. Unregulated heating methods including hot water immersion, microwave heating and tactile temperature estimation are strictly prohibited. Medical thermostats equipped with temperature lock functions are the preferred equipment.Patients must inspect dialysate temperature prior to each infusion and strictly follow standardized warming workflows.Unified warming protocols have been implemented in our hospital and regional home dialysis centers: only medical heaters with a factory maximum temperature limit of 38 °C are permitted, and all simple household heating appliances are banned. All patients and their caregivers must complete specialized warming training before independent home dialysis.Prevention protocols optimized based on this adverse event: one-on-one refresher training is provided for patients with a history of heater malfunctions; on-site verification of warming procedures is conducted during home visits; warning signs regarding peritoneal thermal injury are posted in dialysis zones to reduce recurrence of similar incidents.  2. Hierarchical health education for patients and caregiversPatients and family members are educated on the dual hazards of perceptibly hot peritoneal dialysate:Short-term risks: severe abdominal pain, peritoneal barrier disruption, polymicrobial peritonitis and septic shock;Long-term risks: repeated mild thermal stimulation may induce peritoneal fibrosis and ultrafiltration failure, worsening long-term dialysis outcomes.  3. Intensified follow-up management for high-risk populationsIncrease follow-up frequency for high-risk patients with poor treatment adherence, malnutrition, hypoalbuminaemia, anaemia and impaired immunity. Timely correct underlying comorbidities to improve peritoneal repair capacity and systemic immunity, lowering the risk of severe secondary infection following peritoneal thermal injury.  4. Standardized clinical management for emergency presentationWhen a peritoneal dialysis patient develops abdominal pain immediately after dialysate exchange, clinicians should prioritize enquiries about heater performance, heating methods and tactile sensation of dialysate to screen for suspected peritoneal thermal injury and deliver prompt intervention, blocking progression to lethal sepsis and safeguarding the safety of home dialysis patients. |
